# Acute effects of 150 mg caffeine on subjective, physiological, and behavioral components of anxiety in panic disorder and healthy controls – A randomized placebo-controlled crossover trial

**DOI:** 10.1177/02698811251344692

**Published:** 2025-06-27

**Authors:** Johanna M Hoppe, Johannes Björkstrand, Johan Vegelius, Lisa Klevebrant, Malin Gingnell, Andreas Frick

**Affiliations:** 1 Experimental Cognitive and Affective Neuroscience Lab, Department of Medical Sciences, Uppsala University, Uppsala, Sweden; 2 Department of Psychology, Uppsala University, Uppsala, Sweden; 3 Department of Psychology, Lund University, Lund, Sweden

**Keywords:** Anxiety, caffeine, panic disorder

## Abstract

**Background:**

Caffeine in doses above 400 mg, approximately four cups of coffee, induces panic attacks in 50% of individuals with panic disorder (PD) and elevates anxiety, but it is not known how individuals with PD respond to normally consumed doses or how caffeine interacts with emotional tasks.

**Aims:**

We hypothesized that 150 mg caffeine would increase subjective anxiety (primary outcome) as well as interoceptive attention and anxiety from bodily signals in patients with PD, and more so than in healthy controls (HCs). Additional analyses targeted panic attacks, emotional reactivity, avoidance behavior, and subjective exteroceptive attention.

**Methods:**

Twenty-nine patients with PD and 53 HC with low habitual caffeine consumption (⩽300 mg/week) abstained from caffeine 36 h before receiving 150 mg caffeine or placebo in a double-blind randomized crossover design 2–14 days apart.

**Results:**

Contrary to our hypotheses, caffeine did not increase subjective anxiety, interoceptive attention, or anxiety from interoceptive signals. Only one panic attack was noted, in the PD group after caffeine intake during the emotional reactivity task. In both PD and HC, caffeine increased skin conductance responses to neutral and emotional faces, augmented costly avoidance behavior, and impaired exteroceptive attention. These results indicate that low caffeine doses do not have differential anxiogenic effects in patients with PD and HC at rest, and that they increase arousal and avoidance behavior in both PD and healthy individuals.

**Conclusions:**

In conclusion, we suggest that recommendations for caffeine abstinence for patients with PD should be based on higher doses and ideally on individual assessments.

## Introduction

Panic disorder (PD) is a debilitating psychiatric condition that affects around 5% of the population (Kessler et al., 2005a). It is characterized by re-occurring unexpected panic attacks, that is, sudden onsets of intense fear or discomfort and a range of bodily symptoms, including numbness, dizziness, shortness of breath, and palpitations (American Psychiatric Association, 2013). Patients with PD also suffer from anticipatory anxiety about experiencing new attacks and/or behavioral avoidance of situations that may trigger panic attacks. The etiology of PD is not fully elucidated. Both neurobiological and psychological theories have been articulated (Clark et al., 1997; Gorman et al., 2000; Klein, 1993), and a number of challenge paradigms have been devised to study the underlying neurobiological and psychological components, including caffeine consumption, lactate infusion, CO_2_ inhalation, and behavioral tasks (Charney et al., 1985; Johnson et al., 2014; Klevebrant and Frick, 2022; Pitts and McClure, 1967).

Caffeine generally has positive effects on alertness, wakefulness, and mood in healthy adults when consumed in doses up to 300 mg (roughly equivalent to three cups of coffee; McLellan et al., 2016). Yet, caffeine has also been observed to have anxiogenic and panicogenic properties in higher doses or in vulnerable populations such as individuals with PD (Charney et al., 1985). Supporting the anxiogenic acute effect of caffeine, we showed in a recent meta-analysis of placebo-controlled studies that about half of individuals with PD and 2% of healthy control (HC) participants experienced a panic attack after oral intake of caffeine doses exceeding roughly four to five cups of coffee (400–750 mg) and that these doses also increased subjective anxiety (Klevebrant and Frick, 2022). However, studies investigating the anxiogenic effects of caffeine in PD are few and have methodological limitations. First, caffeine effects in PD have typically been investigated using high caffeine doses (>400 mg), and studies using doses more equivalent to everyday serving sizes, for example, 150 mg, are lacking (Klevebrant and Frick, 2022; Vilarim et al., 2011). Examining the impact of lower caffeine doses is essential to inform clinical recommendations regarding caffeine intake and to enhance understanding of the biological mechanisms underlying caffeine-induced anxiety in PD. Indeed, moderate caffeine doses, such as 150 mg, have been reported to elicit anxiety in healthy individuals with genetic vulnerability (Alsene et al., 2003; Childs et al., 2008). Also, anxiogenic effects of caffeine have almost exclusively been assessed at rest, hampering the ecological validity of findings as real-world settings often contain emotional demands after caffeine consumption.

The skin conductance response (SCR) is a measure of sympathetic nervous system activation often used to index physiological arousal or orienting response to novel stimuli (Boucsein et al., 2012). In both PD and healthy, caffeine increases skin conductance levels and the orienting response (Davidson and Smith, 1991; Quinlan et al., 1997), but studies examining the effect of caffeine on emotional reactivity (ER) using physiological outcomes are largely lacking. The only exception, to the best of our knowledge, is the study by Totten and France (1995), who administered 200 mg caffeine and placebo to individuals with PD with mild symptoms and HC in a randomized crossover design prior to exposure to emotional, physical, and cognitive stressors. They reported caffeine-related increases in skin conductance levels during the stressors but found no support for greater vulnerability in the PD group.

A clinically important and functionally impairing characteristic of PD is maladaptive avoidance behavior. Initially, this is often expressed as avoidance of situations or activities associated with previous panic attacks. However, the avoided situations often generalize to similar situations and activities, leading to more extensive avoidance patterns and impairment in everyday life. Avoidance is specifically maladaptive in situations where it leads to the loss of a reward, sometimes called costly avoidance (Hoppe et al., 2022; Pittig et al., 2021). For instance, avoiding valued situations such as going to the cinema or gym because of fear of suffering from a panic attack both leads to a loss of rewarding experiences and robs the individual of the chance to learn that panic attacks indeed are not dangerous. These situations can be conceptualized as approach-avoidance conflicts and are abundant in real life where they interfere with goals in life. We and others have used experimental approach-avoidance conflict tasks (AACTs) to investigate maladaptive avoidance, showing that high trait-anxiety and patients with anxiety disorders, including PD, display more costly avoidance (Hoppe et al., 2022; Pittig et al., 2021). As outlined above, there are only few studies combining caffeine challenge with emotional tasks in PD, despite the importance of this combination to test for interactive effects between caffeine and emotional demands on anxiety and panic attacks.

Cognitive theories of PD posit that panic attacks, in part, arise from misinterpretations of normal bodily symptoms as threatening, which elicits more symptoms and creates a feedback loop that intensifies anxiety (Clark, 1986; Clark et al., 1997). In the case of caffeine, increased blood pressure or heart rate following caffeine consumption might, for instance, be perceived as a threat, such as signs of a heart attack, potentially triggering a panic attack. Thus, some of the excitatory effects of caffeine on arousal may, in sensitive individuals, lead to increased anxiety through cognitive appraisal of them as a threat. However, caffeine’s impact on interoceptive processing has not been directly investigated, nor has the role of interoceptive processing in the anxiogenic and panicogenic effects of caffeine in PD.

To address the knowledge gaps mentioned above, we conducted a randomized, placebo-controlled, double-blind study using a crossover design to investigate the effects of 150 mg caffeine (vs placebo) on subjective anxiety (primary outcome), panic attacks, physiological ER, avoidance behavior, and interoceptive processing in individuals with PD (vs HC). We hypothesized that in patients with PD, caffeine will (1) be anxiogenic, (2) increase attention to interoceptive stimuli, and (3) increase anxiety associated with experiencing interoceptive stimuli, and that these effects will be stronger in patients with PD than in HC. We also expected a higher occurrence of panic attacks, as well as more attention to interoceptive stimuli and anxiety from such stimuli, in patients with PD than in HC in both caffeine and placebo conditions. Exploratory analyses targeted ER, approach-avoidance behavior, and impairment in exteroceptive attention from interoceptive signals.

## Methods

### Participants

Patients with PD and HC were recruited from advertisements on social media. The recruitment procedure included an initial web screening and, if eligible, a telephone interview incorporating a diagnostic assessment using the Mini international neuropsychiatric interview (Sheehan et al., 1998). A clinical psychologist or psychology student under supervision conducted the telephone interview and determined the diagnostic status. For eligibility in the PD group, participants had to meet the criteria for PD diagnosis according to the fifth version of the Diagnostic statistical manual of mental disorders (American Psychiatric Association, 2013). Due to high levels of comorbidity among individuals with PD (Kessler et al., 2005b), comorbidity with other mental disorders (e.g., mild-moderate depression or other anxiety disorders) did not constitute an exclusion criterion. However, participants with a current or history of severe psychiatric disorder (e.g., psychotic disorder) were excluded. Inclusion in the HC group required the absence of current or history of mental disorders. Participants in both groups had to be at least 18 years old and have a caffeine consumption not exceeding 300 mg per week (corresponding to approximately three to four cups of coffee per week). This relatively low weekly caffeine limit was set to avoid confounding effects on results due to caffeine tolerance and the potential abstinence symptoms elicited by the 36-h washout period before each session and subsequent withdrawal-reversal in the caffeine condition (Rogers et al., 2010). Habitual caffeine usage was assessed through self-reported amounts of weekly consumption of coffee (e.g., espresso, instant coffee), tea (black, green, red, herbal), soft drinks (e.g., cola and energy drinks), chocolate, medications, and other sources of caffeine, and was calculated according to the guidelines of the European Food Safety Authority (EFSA Panel on Dietetic Products and Nutrition and Allergies, 2015). Self-reported caffeine consumption was assessed in the web screening and confirmed during the telephone interview. Included participants completed the self-report form of the Panic Disorder Severity Scale-self-report version (PDSS-SR) online (Houck et al., 2002; Svensson et al., 2019). The PDSS-SR consists of seven items that assess the frequency, distress, and consequences of panic attacks during the last week. Each item is scored on a 5-point scale (0–4), and the total score is between 0 and 28, with higher scores indicating more severe symptoms.

Exclusion criteria for both the PD and HC groups consisted of ongoing treatment with psychotropic medication or discontinued within 2 months, current substance disorder, habitual nicotine use, somatic and neurological conditions (e.g., hypertension and heart condition), or ongoing treatments that may confound the results, uncorrected vision or hearing impairment, and pregnancy.

The study was approved by the Swedish Ethical Review Authority (ID: 2019-06451 and 2020-02260) and conducted in accordance with the Helsinki Declaration. All participants provided written informed consent. Upon completing the two sessions, participants received a gift card worth 500 SEK (approximately 50 USD). The study was preregistered at ClinicalTrials.gov (ID: NCT05261594).

### Design and intervention

The current study was a double-blind, randomized, placebo-controlled trial using a crossover design, including two groups (PD and HC). The study entailed two sessions, >36 h apart, in which participants received identical capsules containing 150 mg caffeine or a placebo substance (microcrystalline cellulose) in randomized order.

### Randomization

A statistician, independent from the project, carried out equal randomization, 1:1, for the two groups using block randomization method with a block size of four and the package *blockrand* (https://CRAN.R-project.org/package=blockrand) in R software version 4.2.2 (R Core Team, 2022a). Participants and the experiment leader were blinded to sequence order (caffeine-placebo vs placebo-caffeine). Unblinding was done for all participants after the completion of data collection.

### Outcome measures

The study’s primary outcome measure consisted of caffeine-induced change (caffeine vs placebo) in subjective anxiety in individuals with PD compared to HC. Here, we also report the following secondary outcomes: (1) *panic attacks*, (2) *physiological ER using* SCRs, (3) *avoidance behavior* in an AACT, and (4) *interocepti*ve *processing* (attention, associated anxiety, and impaired exteroceptive attention).

#### Subjective anxiety

**Figure 1.**
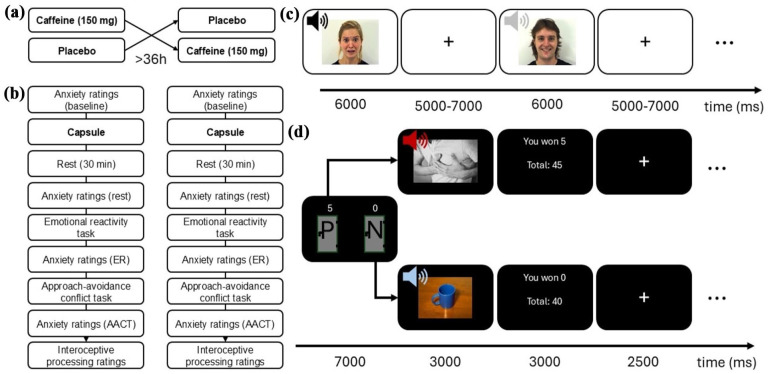
Study overview. (a) The study used a crossover design in which participants underwent the same experimental procedure twice, one with 150 mg caffeine and one with placebo. The order in which participants received the caffeine/placebo capsule was randomized and blinded to both participants and the experiment leader. (b) The two sessions were identical in structure except for the capsule contents, with ratings of anxiety interspersed with tasks, and interoceptive processing ratings at the end. (c) The emotional reactivity (ER) task consisted of 15 trials with 5 faces displaying fear, joy, and neutral expression for 6000 ms started simultaneously with corresponding sounds of a scream, laughter, or humming for 1500 ms, interspersed with crosshairs for 5000–7000 ms. Skin conductance was recorded from the non-dominant hand. (d) The approach-avoidance conflict task (AACT) trial structure. The AACT included 32 trials where participants chose between two doors. One of the doors was always a neutral (N), which corresponded to a neutral image and sound. The other two doors, which appeared on half of the trials each, were a panic (P), corresponding to panic-related images (e.g., crowds or chest pain) and sounds (e.g., heartbeat or panting), and an aversive (O) door, corresponding to generally aversive images and screams. Choosing the panic or aversive door was rewarded with differing amounts of points on each trial (0, 1, 5, and 20 points), and the neutral door was never rewarded (0 points).

Subjective anxiety was measured with self-report on a numerical rating scale ranging from 0 (no anxiety) to 100 (extreme anxiety). It was assessed at four time points (Figure 1): (1) at baseline (before capsule intake), (2) after 30 min of rest after capsule intake, (3) after completion of the ER task (approximately 40 min after capsule intake), and (4) after completion of the AACT (approximately 55 min after capsule intake). The 30-min resting time was chosen to allow for adequate rise in plasma caffeine concentration (Fredholm et al., 1999) and facilitate comparisons to prior findings as this is one of the most frequently used resting times in previous caffeine-challenge studies in PD (Klevebrant and Frick, 2022).

#### Panic attacks

The occurrence of panic attacks during each session was coded on a dichotomous scale (yes or no) based on self-reported panic attacks in conjunction with observations of panic symptoms made by the experiment leader.

#### Emotional reactivity

Participants underwent an ER task consisting of presentations of fearful, happy, and neutral facial expressions and congruent vocal sounds (fearful–scream, happy–laughter, neutral–humming [the sound “hmm”]). SCR to fearful, happy, and neutral conditions were used as outcome.

Static images of faces were drawn from the Amsterdam dynamic facial expression set (Van Der Schalk et al., 2011), and sounds were downloaded from *freesoundeffects.com* and *pixabay.com.* Please refer to Supplemental Table S1, for further details on experimental stimuli. Before the presentation of the first experimental stimulus, a habituation stimulus (image of a flower and sound of dripping water) was displayed for 6000 ms. The experimental procedure included five trials of each emotion condition (fearful, happy, neutral), presented in randomized order, stratified by sex (female and male) and ethnicity (North European and Mediterranean) of the faces. The onset of each image and the corresponding sound was simultaneous. Images were displayed for 6000 ms, and sounds were played for 1500 ms. A black crosshair on a white background was presented on the screen for 5000–7000 ms (on average 6000 ms) between each experimental stimulus. Different experimental stimuli were used in sessions 1 and 2 to reduce habituation effects. The experimental procedure was programmed in E-prime 2.0 (Psychology Software Tools, Pittsburgh, PA, USA). See Figure 1(c) for an overview of the ER task.

Skin conductance was measured during the ER task using BIOPAC MP 160 (BIOPAC Systems, Goleta, CA, USA) and two disposable 11-mm Ag/AgCl-electrodes (EL509) with isotonic electrolyte gel, attached to the hypothenar eminence of the non-dominant hand. The skin conductance signal was sampled at 100 Hz with a high-pass hardware filter of 0.05 Hz and was analyzed using in-house scripts in Matlab R2021a (Mathworks Inc., Natick, MA, USA). The skin conductance signal was first median-sampled using a 10-sample window length, low-pass filtered at 5 Hz, and downsampled to 10 Hz. SCRs to each trial were then calculated using an automated baseline correction method by subtracting the mean of the skin conductance signal in the baseline time window 500 ms after stimulus onset from the maximum of the skin conductance signal during the response window 1–5 s after stimulus onset. SCRs < 0.01 μS were scored as 0 and SCRs > 5 μS were treated as physiologically implausible and removed from further analyses. SCRs were square-root transformed and partitioned according to stimulus and emotion condition (habituation; fearful, happy, and neutral faces + sounds).

#### Avoidance behavior

Avoidance behavior of generally aversive and panic-related stimuli was assessed in the lab using a modified version of the AACT (Hoppe et al., 2022). The AACT puts the participants in an incentive conflict situation where they have to choose between receiving a reward for viewing an aversive image + sound or receiving no reward and viewing neutral image + sound. Costly avoidance behavior was defined as choosing the neutral option in the AACT, that is, forfeiting the reward by avoiding the aversive/panic stimuli.

The AACT was implemented as a game where participants could collect points by approaching generally aversive or panic-related stimuli. The task consisted of 32 trials where the participant chose between one of two doors. The task included three types of doors marked with different letters signaling their content, “O” (generally aversive), “P” (panic-related), and “N” (neutral). One of the doors in each trial was always the neutral door. The other door was either the generally aversive or panic door, distributed evenly throughout the trials in a randomized order. The number of points rewarded if choosing the door was displayed above each door. The generally aversive images depicted bodily injuries and excrements accompanied by the sound of a fearful female scream. Panic-related stimuli included images of people in crowded places, persons displaying chest pain or being passed out paired with sounds of crowds, heart beating or panting/hyperventilation, respectively. The neutral images consisted of neutral objects (e.g., a hand holding an apple, and a clean bathroom) presented together with the rippling water or paper folding. Neutral doors were always rewarded with 0 points, and the generally aversive and panic-related doors were rewarded with 0, 1, 5, or 20 points, varying per trial. The doors were presented until the participant made a decision or for a maximum of 7000 ms. If the participant did not choose a door during that time, they would see the neutral image + sound and receive 0 points. After choosing a door, participants were first presented with the corresponding image + sound combination for 3000 ms and then a screen presenting the number of points acquired on the last trial and the total number of points for 3000 ms. Between each trial, a fixation cross was presented for 2500 ms. Sessions 1 and 2 used different stimuli to reduce habituation effects. Notably, points were not linked to a monetary reward. However, previous research, including our own work (Hoppe et al., 2022), has demonstrated that hypothetical rewards can effectively modulate approach and avoidance behaviors (e.g., Aupperle et al., 2011; Pittig, 2019; Pittig et al., 2018), indicating that receiving points alone is a sufficient incentive to influence decision-making processes. The experimental procedure was programmed in E-prime 2.0 (Psychology Software Tools, Pittsburgh, PA, USA); see Figure 1(d). For analyses in the current study, we collapsed across trial types (panic and aversive) and removed trials with 0 reward points for choosing the aversive or panic door to focus on the incentive-conflict trials (Hoppe et al., 2022).

#### Interoceptive processing

Interoceptive processing was evaluated for the ER and AACT using self-reported ratings of (1) interoceptive attention to bodily sensations associated with panic attacks (e.g., heartbeat and respiration; 0%–100% attention), (2) anxiety elicited by interoceptive signals of bodily sensations (0–100; no anxiety–extreme anxiety), and (3) impaired exteroceptive attention, that is, how much interoceptive attention impaired their ability to focus on the task (0%–100% impaired exteroceptive attention). Impaired exteroceptive attention was not included in the preregistration, but added prior to data collection and included here as an exploratory measure. All interoceptive outcome measures were assessed at the end of each session and included retrospective questions about interoceptive processing during the ER and the AACT.

### Procedure

**Figure 2.**
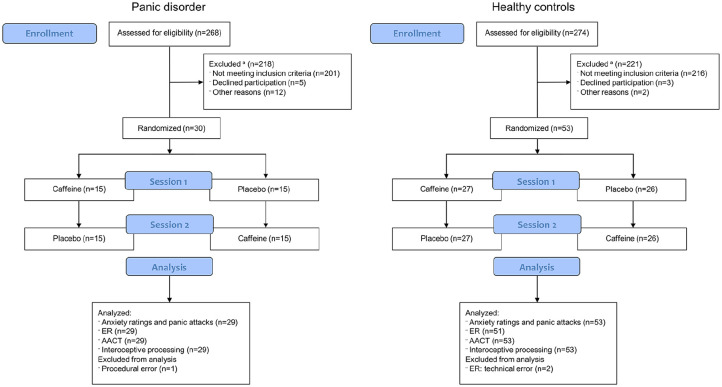
Consort flowchart. ^a^Detailed information about reasons for exclusions during enrollment for both groups is presented in Supplemental Table S2.

The study entailed two sessions following the same procedure, except for the collection of saliva samples in session 1, used for genotyping (not reported here). Each session lasted about 2 h and entailed 30 min of rest after capsule intake, followed by three different computerized experimental tasks. Note that data from the third task (effort-for-reward task) will be reported elsewhere. See Figure 2 for a study overview (task 3 excluded). A minimum of 36 h between sessions was set as a washout period.

Participants were instructed to refrain from caffeine, alcohol, and nicotine for 36 h and fast for 3 h before each session. Upon arrival, compliance with restrictions was confirmed verbally, and baseline anxiety ratings were completed. Participants were then given a capsule along with instructions that the capsule could contain either 150 mg of caffeine, corresponding to approximately 1.5–2 cups of coffee, or a placebo substance. After capsule intake, participants rested for 30 min, during which they were allowed to read magazines but not use their phones. After the resting period, participants completed anxiety and panic attack ratings. Subsequently, the ER task was administered while measuring SCRs. Before starting the task, the sound volume used in the experimental procedure was calibrated using a two-step procedure to be unpleasantly loud but not hurt their hearing, to increase the aversiveness of the fear–scream combination. First, participants listened to a recording of the reading of a neutral text and set their sound level to their preferred listening volume. Then, they listened to an alarm sound and set the volume markedly unpleasant but bearable. Participants were instructed to sit still and pay attention to the screen during the task. Visual stimuli were presented on a 23.8″ computer screen, and over-ear headphones were used for audio stimuli. Upon completion of the task, participants rated anxiety and panic symptoms again. Participants then completed the AACT using the same computer screen and headphones, and again rated anxiety and panic symptoms. Finally, participants rated interoceptive processing variables.

### Statistical analyses

All statistical analyses were performed in R 4.3.1 (R Core Team, 2022b). Linear mixed-effects models were implemented using the package lmerTest (Kuznetsova et al., 2017) for subjective anxiety, ER, and interoceptive processing. For avoidance behavior, choices in the AACT were modeled trial-wise using generalized linear mixed-effects models with a binomial family and logistic link function using the lme4 package (Bates et al., 2015). Both standardized (β) and unstandardized (*b*) coefficients are reported for all linear mixed-effects models, and odds ratios (ORs) for generalized linear mixed-effects models. Standardized coefficients and ORs provide effect size measures comparable between models, and the unstandardized coefficients provide information on the same scale as the used outcome and predictors.

We first tested for the main effects of substance (caffeine or placebo) and group (PD or HC) by fitting mixed-effects models, including participant as random intercept and substance and group as fixed effects. For subjective anxiety, time point (baseline, rest (30 min after capsule intake), ER task, and AACT) was also added as fixed effect, and for ER, emotion (neutral, happy, and fear) was added. To test for differential effects of caffeine in the PD and HC groups, the substance × group interaction was added to the initial models in a second step. Statistical tests of significance of the fixed effects used Satterthwaite’s method. Based on the differences in sex distribution and habitual caffeine consumption, we conducted additional sensitivity analyses, adding these variables as well as the sequence order (caffeine-placebo or placebo-caffeine) to all models. Differences in the occurrence of caffeine-induced panic attacks (caffeine or placebo) and group differences (PD or HC) were assessed with a Chi-square test for the resting condition (30 min) and ER and AACT separately.

## Results

### Participants

Thirty individuals with PD and 53 HC were recruited through advertisements on social media, included in the study, and randomized to either the sequence caffeine-placebo or placebo-caffeine. One patient assigned to the sequence placebo-caffeine was excluded due to procedural error (rested only 20 min after capsule intake), leaving 29 patients with PD and 53 HC to analyze (Table 1). Age was similar in both groups, whereas the proportion of women, weekly habitual caffeine consumption, and ratings on the PDSS-SR were higher in the PD group. Data collection took place between March 2022 and March 2023. See Figure 2 for Consort flowchart.

**Table 1.** Participant characteristics.

Measure	PD (*n* = 29)	HC (*n* = 53)	Statistic	*p*
Age years, *M* (SD)	24.2 (5.1)	24.7 (6.4)	*t*(80) = 0.34	0.734
Range	19–41	18–47		
Sex, *n* (%) women	25 (86%)	33 (62%)	χ^2^(1) = 4.10	0.043
Weekly caffeine intake mg, *M* (SD)	133 (108.9)	84.3 (89.9)	*t*(80) = 2.19	0.031
Range	0–300	0–300		
PDSS-SR, *M* (SD)	9.3 (6.1)	0.5 (1.8)	*t*(80) = 9.81	<0.001
Panic attacks last week, *M* (SD)	0.4 (0.5)			
Comorbidity				
Agoraphobia	11			
Depression	7			
Generalized anxiety disorder	4			
Social anxiety disorder	1			

HC: healthy control; PD: panic disorder; PDSS-SR: panic disorder severity scale – self report.

### Primary outcome. Subjective anxiety

**Figure 3.**
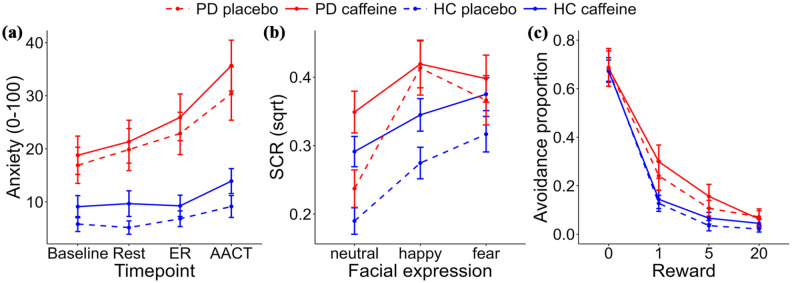
Subjective anxiety, physiological response, and behavioral avoidance. (a) Subjective anxiety was assessed before capsule intake (baseline), after 30 min of rest, and after the emotional reactivity (ER) and approach-avoidance conflict tasks (AACT). Patients with panic disorder (PD) had overall higher anxiety levels than healthy controls (HCs), and anxiety increased over the session, but 150 mg caffeine had no effect on anxiety ratings or moderated the effect of group (PD; HC) or task on anxiety ratings. (b) Caffeine (150 mg) induced general higher skin conductance responses (SCR) across stimuli, with no differential effect between PD and HC or between neutral and emotional stimuli. SCR was higher for emotional than neutral stimuli. (c) Costly avoidance in the approach-avoidance conflict task (AACT) was higher in PD than HC, increased with 150 mg caffeine, and decreased with higher rewards. Error bars denote standard errors.

We fitted a linear mixed-effects model with subjective anxiety (0–100) as outcome, participant as random intercept, and group (PD, HC), substance (caffeine, placebo) and time point (baseline, rest, ER, and AACT) as fixed effects. The PD group reported generally higher subjective anxiety than HC and anxiety was also higher during the caffeine visits than placebo visits (including the baseline measures) and for the ER and AACT compared to baseline (Table 2, Figure 3, and Supplemental Table S3). We then entered all possible interaction effects between these fixed factors and found only that subjective anxiety increased more from baseline to the AACT in the PD group than in the HC (standardized β = 0.532, unstandardized *b* = 10.197, *t* = 2.74, *p* = 0.006), all other *p*’s > 0.17. Thus, contrary to our hypothesis, we found no support for an effect of 150 mg caffeine on change in subjective anxiety from baseline to rest or to ER or AACT, that is, our primary outcome measure.

**Table 2.** Mixed effects models of subjective anxiety, emotional reactivity, and avoidance behavior. Standardized (β) and unstandardized (*b*) coefficients are reported, and odds ratios (OR) for avoidance behavior. Placebo session, healthy controls, baseline anxiety ratings, neutral emotion are reference levels.

Model	β	*b*	SE	*t*	*p*
Anxiety ratings					
Substance: caffeine	0.181	3.467	0.900	3.853	<0.001
Group: PD	0.802	15.371	3.184	4.827	<0.001
Time point: rest (30 min)	0.049	0.945	1.271	0.743	0.458
Time point: ER	0.141	2.695	1.271	2.120	0.034
Time point: AACT	0.419	8.025	1.274	6.301	<0.001
Baseline anxiety ratings					
Substance: caffeine	0.175	2.793	1.743	1.602	0.113
Group: PD	0.649	10.373	3.010	3.446	0.001
Emotional reactivity (SCR)					
Substance: caffeine	0.143	0.055	0.014	4.039	<0.001
Group: PD	0.163	0.063	0.048	1.330	0.187
Emotion: fear	0.257	0.100	0.017	6.020	<0.001
Emotion: happy	0.229	0.089	0.017	5.367	<0.001
Avoidance behavior	OR	*b*	SE	*Z*	*p*
Model 1					
Substance: caffeine	0.652	0.428	0.129	3.322	<0.001
Group: PD	0.239	1.423	0.642	2.228	0.025
Model 2					
Substance: caffeine	0.718	0.331	0.141	2.350	0.019
Group: PD	0.292	1.231	0.643	1.913	0.056
Interoceptive attention	1.005	−0.005	0.006	0.870	0.384
Interoceptive anxiety	1.004	−0.004	0.008	0.445	0.657
Impaired exteroceptive attention	0.975	0.025	0.006	4.195	<0.001

AACT: approach-avoidance conflict task; ER: emotional reactivity; PD: panic disorder.

We then conducted a sensitivity analysis using similar mixed-effects models but focusing only on the baseline measure of anxiety (Table 2 and Figure 3). Patients with PD had higher baseline anxiety, but we found no support for higher baseline anxiety for caffeine sessions compared to placebo sessions. We performed additional sensitivity analyses, including weekly caffeine consumption, sex, and sequence order (caffeine-placebo or placebo-caffeine), to the models without finding support for the contribution of these variables to subjective anxiety or that they changed the pattern of results (Supplemental Table S4).

### Panic attacks

Only one panic attack was observed in the entire study, and this was during the ER in a patient with PD after caffeine intake.

### Emotional reactivity

ER was examined using SCR to neutral, fearful, and happy facial expressions + corresponding sounds in the ER task. Due to technical errors, SCR data was missing for 1 patient for the caffeine session and 2 HCs each for the caffeine and placebo sessions, leaving 29 in the PD group and 51 in the HC group with at least 1 session. A linear mixed-effects model with SCR as outcome, participant as random intercept, and substance (caffeine, placebo), group (PD, HC) and emotion (neutral, fear, happy) as fixed effects showed that caffeine induced a general increase in SCR and that SCRs were higher to both fearful and happy faces than neutral stimuli. No interaction effects were detected (*p*’s > 0.06), indicating that the effects of caffeine did not differ between stimuli or groups (Table 2 and Figure 3).

We then performed sensitivity analyses, including weekly caffeine consumption, sex, and sequence order, to the original model. The pattern of results remained, with the addition of sex but not weekly caffeine consumption or sequence order contributing to the ER (Supplemental Table S4).

### Avoidance behavior

Avoidance behavior was assessed with the AACT. Behavioral data was missing for 1 patient for the caffeine session; thus, we included all 29 patients and 53 HC with at least 1 session in the analyses. Generalized linear mixed effects models were used to analyze costly avoidance behavior. We first tested effects of group (PD, HC) and substance (caffeine, placebo) and found higher behavioral avoidance in the PD group and that caffeine intake increased levels of costly avoidance (Table 2 and Figure 3). No interaction effect was detected (*p* = 0.538), indicating that caffeine-induced changes in behavioral avoidance were not larger in patients than in HC. We then added interoceptive processing variables to the model. Costly avoidance behavior was positively related to impaired exteroceptive attention during the AACT, but not to interoceptive attention or anxiety from interoceptive signals.

Sensitivity analyses were performed by adding weekly caffeine consumption, sex, and sequence order to the models. Neither of the covariates contributed to behavioral avoidance, and the general pattern of results remained, although the effect of group was slightly reduced and could no longer be detected according to the *p* < 0.05 criteria (Supplemental Table S5).

### Interoceptive processing

Six linear mixed-effects models were conducted, one for each task and the three interoceptive variables. All models included participant as random intercept and substance (caffeine or placebo) and group (PD or HC) as fixed effects. Results indicated a similar pattern of effects across both the ER and AACT. Caffeine caused impaired exteroceptive attention during both the ER and AACT. Patients with PD reported greater attention to interoceptive signals, experienced more anxiety from such signals, and reported more impaired exteroceptive attention during the ER and AACT compared to HCs (Tables 3 and 4). No interactions between caffeine and group were detected (*p*s > 0.13), indicating similar effects of caffeine in both groups.

**Table 3.** Mean and standard deviations of interoceptive ratings.

Interoceptive processing	PD (*n* = 29)		HC (*n* = 53)	
	Placebo	Caffeine	Placebo	Caffeine
	*M* (SD)	*M* (SD)	*M* (SD)	*M* (SD)
Attention to bodily signals				
ER	38.8 (27.4)	42.9 (30.5)	28.0 (24.7)	32.3 (23.4)
AACT	38.8 (25.7)	50.0 (28.9)	33.1 (28.3)	34.4 (26.1)
Anxiety from bodily signals				
ER	25.3 (27.7)	36.1 (32.5)	3.8 (12.4)	5.1 (15.0)
AACT	33.0 (32.2)	40.2 (27.3)	9.0 (18.8)	11.4 (18.9)
Impaired exteroceptive attention				
ER	23.4 (21.3)	34.4 (31.6)	10.4 (14.6)	17.7 (22.5)
AACT	26.8 (26.0)	38.5 (28.5)	12.6 (18.6)	18.2 (21.3)

AACT: approach-avoidance conflict task; ER: emotional reactivity; HC: healthy controls; PD: panic disorder.

**Table 4.** Mixed effects models for interoceptive processing. Results from linear mixed effects models of participants’ ratings of attention to, anxiety from, and impaired exteroceptive attention from interoceptive signals during the emotional reactivity (ER) and approach-avoidance conflict task (AACT). Standardized (β) and unstandardized (*b*) coefficients are reported. The placebo session and healthy controls are reference levels.

Model	β	*b*	SE	*t*	*p*
Interoceptive attention					
ER					
Substance: caffeine	0.155	4.060	3.289	1.235	0.221
Group: PD	0.407	10.674	5.058	2.110	0.038
AACT					
Substance: caffeine	0.155	4.297	3.376	1.273	0.207
Group: PD	0.371	10.260	5.365	1.912	0.060
Interoceptive anxiety					
ER					
Substance: caffeine	0.178	4.335	2.789	1.554	0.124
Group: PD	1.086	26.397	3.980	6.632	<0.001
AACT					
Substance: caffeine	0.142	3.768	2.861	1.317	0.192
Group: PD	0.998	26.408	4.589	5.754	<0.001
Impaired exteroceptive attention					
ER					
Substance: caffeine	0.353	8.237	3.097	2.660	0.010
Group: PD	0.647	15.099	4.089	3.693	<0.001
AACT					
Substance: caffeine	0.306	7.468	3.082	2.423	0.017
Group: PD	0.704	17.195	4.312	3.988	<0.001

AACT: approach-avoidance conflict task; ER: emotional reactivity; HC: healthy controls; PD: panic disorder.

Sensitivity analyses were conducted by adding weekly caffeine consumption, sex, and sequence order to the original models. For interoceptive attention for both tasks, we no longer detected a contribution of group (Supplemental Table S6). Weekly caffeine consumption contributed positively to the ratings that bodily signals caused anxiety and impaired exteroceptive attention during the AACT, but not during the ER. Neither sex nor sequence order contributed to the interoceptive processing outcomes.

## Discussion

In this double-blind, randomized, placebo-controlled, crossover administration of 150 mg caffeine, we found no support for our primary hypothesis that acute caffeine administration increases subjective anxiety (i.e., we detected no substance × time point interaction on subjective anxiety). This lack of anxiogenic effect from 150 mg caffeine was evident both during the 30-min rest period after caffeine consumption and during exposure to aversive images and sounds in the ER and AACTs. Patients with PD had higher anxiety than HC in general, but we detected no differential effect of caffeine between groups, contradicting our primary hypothesis. Our findings are in line with the results of a study by Adan et al. (2008), which found no significant effects of 100 mg of caffeine on mood compared to decaffeinated coffee in healthy participants. Only one panic attack was noted in the study, which was in a patient with PD in the caffeine session during the ER task. Overall, these results from 150 mg caffeine are in contrast to previously reported acute effects of higher doses of caffeine (>400 mg), where a panic attack was experienced by about half of the patients with PD together with an increased subjective anxiety during rest (Klevebrant and Frick, 2022). Hence, we conclude that 150 mg caffeine, corresponding to 1.5–2 cups of coffee, generally is not anxiogenic or panicogenic, neither in PD nor in non-clinical individuals, not even when combined with emotional and aversive images and sounds. Analyses of secondary outcomes revealed that 150 mg caffeine increased ER in the form of physiological responses to neutral and emotional faces, amplified costly avoidance behavior, and led to greater subjectively perceived impairment in exteroceptive attention due to increased focus on interoceptive signals. These effects of caffeine were similar across groups, and we could not detect increased vulnerability in patients. Furthermore, we detected no acute effects of caffeine on interoceptive attention to bodily signals or anxiety from such signals. Together with our recent meta-analysis showing anxiogenic and panicogenic effects of high doses of caffeine, the current findings support dose-related effects of caffeine on anxiety and panic attacks in patients with PD and that normal serving sizes of caffeine are, from an anxiogenic perspective, generally safe to consume for patients with PD.

Caffeine increased ER to neutral and emotional faces, as indexed by SCRs. This is in accordance with caffeine’s arousing effects and activation of the sympathetic nervous system (Corti et al., 2002). However, and similar to the previous study by Totten and France (1995), the effects of caffeine did not differ between patients with PD and HCs. Our results further corroborate that caffeine doses corresponding to 1–2 cups of coffee increase sympathetic nervous activity during emotional challenge without separating patients with PD from healthy individuals.

Interestingly, caffeine augmented costly avoidance behavior in the AACT. Compared to placebo sessions, participants were more likely to avoid aversive images + sounds, even at the expense of losing potential rewards, after intake of an acute 150 mg dose of caffeine. This finding may have important implications for patients with PD and other anxiety disorders characterized by maladaptive avoidance. For instance, caffeine may amplify maladaptive avoidance, contributing to the maintenance of fear and further hindering individuals in daily life. If caffeine increases costly avoidance, it may also impact exposure therapy by reducing patients’ willingness to engage with feared stimuli or situations, a critical aspect of this treatment.

There are a number of processes underlying the decision to approach or avoid the incentive-conflict situations used in the current study. Cost-benefit calculations are employed to judge the cost of approaching the aversive stimuli against the reward. Caffeine may shift these cost-benefit calculations, increasing perceived costs or reducing perceived rewards. Another potential explanation for the increased avoidance behavior involves the caffeine-induced increase in physiological arousal. The decision to approach or avoid may include predicting the resulting bodily state if one is exposed to the aversive stimulus. Higher arousal levels could alter these predictions and/or influence how individuals perceive their ability to tolerate the bodily state that aversive stimuli may induce. With increased arousal, there may be a smaller buffer for tolerating additional aversive exposure, leading to avoidance behavior. Another possibility is that caffeine reduces the threshold for the intensity of aversive stimuli or situations individuals believe they can handle. Further studies are needed to clarify the mechanisms underlying caffeine-induced costly avoidance.

Although the effect of caffeine on costly avoidance did not differ between patients and controls, the PD group reported a greater increase in anxiety levels than HC during the AACT and were generally more avoidant of aversive stimuli. In line with these group differences, we have recently reported that high levels of trait anxiety are associated with more costly avoidance during a similar AACT (Hoppe et al., 2022). Additionally, others have shown that patients with anxiety disorders, including PD, exhibit more costly avoidance (Pittig et al., 2021). Taken together, the findings corroborate the view that avoidance behavior is a central component of anxiety disorders, maintaining anxiety symptoms and preventing individuals from learning that the situations (and symptoms) do not lead to the catastrophes they fear.

Apart from the effects of caffeine, we also examined differences in outcome measures between patients with PD and HCs. Patients had higher subjective anxiety levels, exhibited more costly avoidance behavior, and were more affected by interoceptive signals than controls. However, at this dosage, we did not find evidence that caffeine moderates interoceptive attention or heightens anxiety levels caused by attention to bodily sensations during emotional tasks.

The study has some limitations that should be noted. The target sample size of *n* = 50 for the PD group was not reached due to difficulties in recruiting participants fulfilling eligibility criteria (12% inclusion rate; see Supplemental Table S2). The most common cause for exclusion was not fulfilling diagnostic criteria for PD (52%), for example, reporting panic attacks associated with another mental disorder (e.g., social anxiety, posttraumatic stress disorder) or absence of panic-related fear or behavioral change. The second most common exclusion criterion was exceeding the maximum weekly caffeine intake limit of 300 mg (22%). The limited sample size may reduce the ability to detect the effects of 150 mg of caffeine on the outcome measures. Therefore, future studies should aim to replicate these findings in larger samples. Additionally, it could be worth considering whether the eligibility criteria for PD were too stringent. For example, finding a PD sample with no current treatment and a maximum caffeine intake of 300 mg per week might be too restrictive. However, increasing the limit for weekly caffeine consumption could introduce potential biases, as individuals may experience withdrawal symptoms or have increased tolerance. Furthermore, the PD group contained more women and had higher habitual caffeine consumption than the HC group, which may bias the results, although habitual caffeine consumption within our sample was lower than in previous studies examining the effects of caffeine in PD (Klevebrant and Frick, 2022). For instance, Adan et al. (2008) observed that 100 mg of caffeine produced more substantial arousal effects in men compared to women (in healthy individuals). However, when we added sex and habitual caffeine consumption as covariates to the models, the general patterns of results remained, although the contribution of group to the interoceptive processing and behavioral avoidance was slightly reduced. Also, the sessions were not restricted to a specific time of day, which may have introduced circadian variations that influenced the results. Notably, in the study by Adan et al. (2008), no significant effect of 100 mg caffeine on mood was observed, regardless of sex and time of day. Nevertheless, future studies should include a more balanced sex distribution and standardized administration times to enable analyses examining sex differences and the impact of circadian variations on the effects of 150 mg of caffeine on subjective, physiological, and behavioral components of anxiety in PD.

Moreover, the severity level of panic symptoms in the patient group was at the lower end, which may have influenced the results. The sample was drawn from a community population through social media advertisements, and it is possible that a more severe PD population or recruitment from clinics might have revealed different effects of low doses of caffeine in patients compared to HCs. Our operationalization of the interoceptive processing variables was purely based on self-report and future studies should ideally complement these with behavioral tasks of interoception to draw more firm conclusions. Additionally, the questions used to assess subjective measures of interoceptive processing have not been validated in previous studies, and the results should, therefore, be interpreted with caution. Finally, this study only examined the effects of 150 mg of caffeine. Future studies should investigate a broader range of caffeine doses to allow the evaluation of dose-response effects on outcome measures.

In conclusion, our results, in combination with our previous meta-analysis findings of anxiogenic and panicogenic effects of >400 mg caffeine (Klevebrant and Frick, 2022), indicate a dose-response relationship and that caffeine doses in the range of those consumed in everyday life are not anxiogenic in patients with PD or in healthy individuals, but may increase behavioral avoidance. We thus suggest that recommendations for caffeine abstinence for patients with PD should focus on higher doses and, ideally, be based on individual assessments. Our findings also highlight that the relationship between caffeine and avoidance behavior warrants further investigation, as it may influence the maintenance of anxiety disorders and the effectiveness of exposure therapy.

## Supplemental Material

sj-docx-1-jop-10.1177_02698811251344692 – Supplemental material for Acute effects of 150 mg caffeine on subjective, physiological, and behavioral components of anxiety in panic disorder and healthy controls – A randomized placebo-controlled crossover trial Supplemental material, sj-docx-1-jop-10.1177_02698811251344692 for Acute effects of 150 mg caffeine on subjective, physiological, and behavioral components of anxiety in panic disorder and healthy controls – A randomized placebo-controlled crossover trial by Johanna M Hoppe, Johannes Björkstrand, Johan Vegelius, Lisa Klevebrant, Malin Gingnell and Andreas Frick in Journal of Psychopharmacology
