# Supplementary material for: Acute effects of 150 mg caffeine on subjective, physiological, and behavioral components of anxiety in panic disorder and healthy controls – A randomized placebo-controlled crossover trial
Source: J Psychopharmacol. 2025 Jun 27;39(8):836–46. doi: 10.1177/02698811251344692 (PMC12287556; doi:10.1177/02698811251344692)
Supplement: sj-docx-1-jop-10.1177_02698811251344692 – Supplemental material for Acute effects of 150 mg caffeine on subjective, physiological, and behavioral components of anxiety in panic disorder and healthy controls – A randomized placebo-controlled crossover trial [file sj-docx-1-jop-10.1177_02698811251344692.docx]

**Acute effects of 150 mg caffeine on subjective, physiological, and behavioral components of anxiety in panic disorder and healthy controls: a randomized placebo controlled crossover trial**

Supplementary materials

Johanna M. Hoppe^a,b^, Johannes Björkstrand^c^, Johan Vegelius^a,b^, Lisa Klevebrant^a,b^, Malin Gingnell^a^, Andreas Frick^a^

^a^Department of Medical Sciences, Experimental Cognitive and Affective Neuroscience Lab, Uppsala University, Uppsala, Sweden

^b^Department of Psychology, Uppsala University, Sweden

^c^Department of Psychology, Lund University, Sweden

**Supplementary Table S1**

Images and sounds used in the emotional task

| Type | Stimuli set | Stimulus | Source | Original name |
| --- | --- | --- | --- | --- |
| Sound | A and B | Fear sound female | freesoundeffects.com | woscream4 |
| Sound | A and B | Fear sound male | pixabay.com/sound-effects | man-screaming-6373 |
| Sound | A and B | Neutral sound female | pixabay.com/sound-effects | hmms-various-1-7175 |
| Sound | A and B | Neutral sound male | pixabay.com/sound-effects | hmm-oh-6957 |
| Sound | A and B | Happy sound female | soundsnap.com  Wood et al(2017) | p6_c6_2s_desilenced |
| Sound | A and B | Happy sound male | soundsnap.com  Wood et al(2017) | p13_c7_3s_desilenced |
| Image | A | face 1 | ADFES^d^ | North European F03 |
| Image | A | face 2 | ADFES | North European F04 |
| Image | A | face 3 | ADFES | Mediterranean F09 |
| Image | A | face 4 | ADFES | North European M04 |
| Image | A | face 5 | ADFES | Mediterranean M10 |
| Image | B | face 1 | ADFES | North European F01 |
| Image | B | face 2 | ADFES | North European F05 |
| Image | B | face 3 | ADFES | Mediterranean F10 |
| Image | B | face 4 | ADFES | North European M02 |
| Image | B | face 5 | ADFES | Mediterranean M01 |

Note. ^a^All sounds were cut to 1.5 s and normalized to -10 dB. ^b^ Stimulus set A was used in Session 1 and B in Session 2. ^c^International Affective Digitized Sounds 2. ^d^Amsterdam Dynamic Facial Expression Set.

**Supplementary Table S2.**

Overview of exclusion causes for enrollment in the panic disorder (PD) and healthy control groups.

| **Panic group** | **Frequency** | | **Percent** | | |
| --- | --- | --- | --- | --- | --- |
| Included | 30 | | 12.1 | | |
| Excluded | 218 | | 88.0 | | |
| Total assessed for eligibility | 248 | | 100.0 | | |
|  |  | |  | | |
| **Exclusion cause** |  |  | |  |  |
| DSM-5 PD criteria not fulfilled or PD not primary |  | 112 | |  | 51.4 |
| Caffeine > 300 mg |  | 48 | |  | 22.1 |
| Ongoing treatment psychotropic medication |  | 23 | |  | 10.6 |
| Unable to reach |  | 12 | |  | 5.5 |
| Medical condition |  | 6 | |  | 2.8 |
| Nicotine |  | 6 | |  | 2.8 |
| Declined participation |  | 5 | |  | 2.3 |
| Ongoing psychological treatment |  | 3 | |  | 1.4 |
| Other ongoing treatment |  | 2 | |  | 0.9 |
| Substance disorder |  | 1 | |  | 0.5 |
|  |  | |  | | |
| **Healthy controls** |  | |  | | |
| Included | 53 | | 19.3 | | |
| Excluded | 221 | | 80.7 | | |
| Total assessed for eligibility | 274 | | 100.0 | | |
|  |  | |  | | |
| **Exclusion cause** |  | |  | | |
| Caffeine > 300 mg |  | 72 | |  | 32.6 |
| Ongoing or history of psychiatric condition |  | 68 | |  | 30.8 |
| Unable to reach |  | 43 | |  | 19.5 |
| Ongoing treatment |  | 14 | |  | 6.3 |
| Medical condition |  | 13 | |  | 5.9 |
| Nicotine |  | 6 | |  | 2.7 |
| Declined participation |  | 3 | |  | 1.4 |
| Other* |  | 2 | |  | 0.9 |

* Other: For one, the traveling distance to Uppsala was too long, and the other did not have sufficient knowledge of Swedish to understand instructions.

**Supplementary Table S3. Mean and standard deviations of subjective anxiety ratings**

|  | **PD (n=29)** | | **HC (n=53)** | |
| --- | --- | --- | --- | --- |
|  | Placebo | Caffeine | Placebo | Caffeine |
| **Anxiety ratings** | M (SD) | M (SD) | M (SD) | M (SD) |
| Baseline | 16.9 (18.3) | 18.8 (19.4) | 5.8 (10.2) | 9.1 (15.2) |
| Rest (30 min) | 19.9 (21.3) | 21.3 (21.8) | 5.2 (9.1) | 9.7 (17.6) |
| ER | 22.9 (21.4) | 25.9 (23.7) | 6.8 (10.7) | 9.3 (14.7) |
| AACT | 30.4 (27.0) | 35.7 (25.3) | 9.2 (15.1) | 13.9 (17.1) |

AACT: Approach-avoidance conflict task; ER: Emotional reactivity; HC: Healthy controls; PD: panic disorder.

**Supplementary Table S4. Linear mixed effects models of subjective anxiety and physiology including weekly caffeine consumption, sex and sequence order as covariates.** Placebo, group healthy controls, timepoint baseline anxiety ratings, neutral emotion, female, and placebo-caffeine sequence order are reference levels.

| **Anxiety ratings** | ***β*** | ***B*** | ***SE*** | ***t*** | ***P*** |
| --- | --- | --- | --- | --- | --- |
| Substance: Caffeine | 0.181 | 3.467 | 0.900 | 3.853 | <.001 |
| Group: PD | 0.813 | 15.583 | 3.424 | 4.552 | <.001 |
| Timepoint: Rest (30 min) | 0.049 | 0.945 | 1.271 | 0.743 | .458 |
| Timepoint: ER | 0.141 | 2.695 | 1.271 | 2.120 | .034 |
| Timepoint: AACT | 0.419 | 8.025 | 1.274 | 6.301 | <.001 |
| Weekly caffeine consumption | -0.005 | -0.001 | 0.016 | 0.064 | .949 |
| Sex: Male | 0.03 | 0.579 | 3.603 | 0.161 | .837 |
| Sequence: Caffeine-placebo | 0.04 | 0.773 | 3.164 | 0.244 | .808 |
| **Baseline anxiety ratings** | ***β*** | ***B*** | ***SE*** | ***t*** | ***P*** |
| Substance: Caffeine | 0.175 | 2.793 | 1.743 | 1.602 | .113 |
| Group: PD | 0.668 | 10.679 | 3.225 | 3.312 | .001 |
| Weekly caffeine consumption | 0.006 | 0.001 | 0.015 | 0.063 | .950 |
| Sex: Male | 0.079 | 1.266 | 3.394 | 0.383 | .710 |
| Sequence: Caffeine-placebo | 0.119 | 1.900 | 2.980 | 0.638 | .526 |
| **Physiology (SCR)** | ***β*** | ***B*** | ***SE*** | ***t*** | ***P*** |
| Substance: Caffeine | 0.143 | 0.055 | 0.014 | 4.049 | <.001 |
| Group: PD | 0.218 | 0.085 | 0.050 | 1.719 | .090 |
| Emoton: Fear | 0.257 | 0.100 | 0.017 | 6.020 | <.001 |
| Emotion: Happy | 0.229 | 0.089 | 0.017 | 5.367 | <.001 |
| Weekly caffeine consumption | 0.041 | 0.0002 | 0.002 | 0.676 | .501 |
| Sex: Male | 0.323 | 0.125 | 0.052 | 2.410 | .018 |
| Sequence: Caffeine-placebo | 0.563 | 2.184 | 0.046 | 0.479 | .633 |

AACT: Approach-avoidance conflict task; ER: Emotional reactivity; PD: panic disorder.

**Supplementary Table S5. Linear mixed effects models for behavioral avoidance including weekly caffeine consumption, sex, and sequence order as covariates.** Results from generalized linear mixed effects models of participant’s trial-by-trial choice to approach or avoid aversive stimuli for rewards**.** Odds ratios (OR) and unstandardized (b) coefficients are reported. Placebo, group healthy controls, female, and placebo-caffeine sequence order are reference levels.

| **Behavioral avoidance** | ***OR*** | ***b*** | ***SE*** | ***Z*** | ***p*** |
| --- | --- | --- | --- | --- | --- |
| **Model 1** |  |  |  |  |  |
| Substance: Caffeine | 0.652 | 0.428 | 0.129 | 3.325 | <.001 |
| Group: PD | 0.343 | 1.070 | 0.650 | 1.645 | .100 |
| Weekly caffeine consumption | 0.682 | 0.004 | 0.003 | 1.215 | .224 |
| Sex: Male | 2.167 | -0.773 | 0.738 | 1.049 | .294 |
| Sequence: Caffeine-placebo | 0.703 | 0.417 | 0.618 | 0.569 | .569 |
| **Model 2** |  |  |  |  |  |
| Substance: Caffeine | 0.716 | 0.334 | 0.141 | 2.364 | .018 |
| Group: PD | 0.376 | 0.979 | 0.652 | 1.501 | .133 |
| Interoceptive attention | 1.006 | -0.006 | 0.006 | 0.953 | .341 |
| Interoceptive anxiety | 1.003 | -0.003 | 0.008 | 0.415 | .678 |
| Impaired exteroceptive attention | 0.976 | 0.024 | 0.006 | 4.068 | <.001 |
| Weekly caffeine consumption | 0.811 | 0.002 | 0.003 | 0.680 | .497 |
| Sex: Male | 2.346 | -0.853 | 0.733 | 1.164 | .245 |
| Sequence: Caffeine-placebo | 0.687 | 0.375 | 0.603 | 0.622 | .534 |

**Supplementary Table S6. Linear mixed effects models for interoceptive processing including weekly caffeine consumption, sex, and sequence order as covariates.** Results from linear mixed effects models of participant’s ratings of attention to, anxiety from, and impaired exteroceptive attention from interoceptive signals during the emotional reactivity (ER) and approach-avoidance conflict tasks (AACT). Standardized (β) and unstandardized (b) coefficients are reported. Placebo, group healthy controls, female, and caffeine-placebo sequence order are reference levels.

| **Interoceptive attention** | ***β*** | ***b*** | ***SE*** | ***t*** | ***p*** |
| --- | --- | --- | --- | --- | --- |
| **ER** |  |  |  |  |  |
| Substance: Caffeine | 0.148 | 3.877 | 3.291 | 1.178 | .242 |
| Group: PD | 0.324 | 8.490 | 5.394 | 1.574 | .120 |
| Weekly caffeine consumption | -0.015 | -0.004 | 0.025 | 0.153 | .879 |
| Sex: Male | -0.336 | -8.801 | 5.648 | 1.558 | .123 |
| Sequence: Caffeine-placebo | 0.130 | 3.418 | 4.886 | 0.700 | .486 |
| **AACT** |  |  |  |  |  |
| Substance: Caffeine | 0.152 | 4.218 | 3.371 | 1.251 | .215 |
| Group: PD | 0.234 | 6.491 | 5.557 | 1.165 | .248 |
| Weekly caffeine consumption | 0.108 | 0.030 | 0.027 | 1.134 | .261 |
| Sex: Male | -0.362 | -10.009 | 5.908 | 1.694 | .094 |
| Sequence: Caffeine-placebo | 0.107 | 2.971 | 5.166 | 0.575 | .567 |
| **Interoceptive anxiety** | ***β*** | ***b*** | ***SE*** | ***t*** | ***p*** |
| **ER** |  |  |  |  |  |
| Substance: Caffeine | 0.178 | 4.330 | 2.790 | 1.552 | .125 |
| Group: PD | 1.065 | 25.886 | 4.320 | 5.992 | <.001 |
| Weekly caffeine consumption | 0.060 | 0.015 | 0.020 | 0.726 | .470 |
| Sex: Male | 0.035 | 0.841 | 4.484 | 0.188 | .852 |
| Sequence: Caffeine-placebo | -0.069 | -1.673 | 3.900 | 0.429 | .669 |
| **AACT** |  |  |  |  |  |
| Substance: Caffeine | 0.138 | 3.652 | 2.858 | 1.278 | .205 |
| Group: PD | 0.894 | 23.659 | 4.780 | 4.950 | <.001 |
| Weekly caffeine consumption | 0.174 | 0.047 | 0.023 | 2.043 | .045 |
| Sex: Male | -0.068 | -1.810 | 5.068 | -0.357 | .722 |
| Sequence: Caffeine-placebo | 0.100 | 2.640 | 4.431 | 0.596 | .553 |
| **Impaired exteroceptive attention** | ***β*** | ***b*** | ***SE*** | ***t*** | ***p*** |
| **ER** |  |  |  |  |  |
| Substance: Caffeine | 0.347 | 8.093 | 3.097 | 2.613 | .011 |
| Group: PD | 0.561 | 13.077 | 4.381 | 2.985 | .004 |
| Weekly caffeine consumption | 0.093 | 0.022 | 0.021 | 1.063 | .291 |
| Sex: Male | -0.120 | -2.805 | 4.591 | 0.611 | .543 |
| Sequence: Caffeine-placebo | 0.170 | 3.972 | 3.970 | 1.001 | .320 |
| **AACT** |  |  |  |  |  |
| Substance: Caffeine | 0.300 | 7.322 | 3.066 | 2.389 | .019 |
| Group: PD | 0.512 | 12.525 | 4.275 | 2.930 | .005 |
| Weekly caffeine consumption | 0.262 | 0.065 | 0.020 | 3.168 | .002 |
| Sex: Male | -0.267 | -6.531 | 4.531 | 1.442 | .154 |
| Sequence: Caffeine-placebo | 0.026 | 0.625 | 3.964 | 0.158 | .875 |

PD: Panic disorder

REFERENCES

Wood, A., Martin, J., & Niedenthal, P. (2017). Towards a social functional account of laughter: Acoustic features convey reward, affiliation, and dominance. *PLoS ONE*, *12*(8), e0183811. https://doi.org/10.1371/journal.pone.0183811
